# Interhemispheric interactions in visual word recognition: the role of multiple meanings

**DOI:** 10.3389/fpsyg.2025.1591311

**Published:** 2025-07-22

**Authors:** Sangyub Kim, Kichun Nam

**Affiliations:** ^1^Department of Psychology, Chonnam National University, Gwangju, Republic of Korea; ^2^School of Psychology, Korea University, Seoul, Republic of Korea

**Keywords:** semantic processing, lexical decision task, visual half-field paradigm, number of meanings, hemispheric interactions, hemispheric dynamics, visual word recognition

## Abstract

**Introduction:**

The current study investigated the influence of the number of meanings on visual word recognition, with a particular focus on hemispheric dynamics.

**Methods:**

By reanalyzing parafoveal Korean lexical decision data, we examined how words with a high versus low number of meanings affect right visual field advantage (RVFA) and bilateral redundancy gain (BRG).

**Results:**

The words with a greater number of meanings exhibited a stronger RVFA and reduced BRG compared to words with fewer meanings.

**Discussion:**

The findings suggest that the facilitatory effects associated with multiple meanings are more pronounced in the left hemisphere (LH) than in the right hemisphere (RH). Furthermore, the increased lateralization of processing within the LH appears to diminish the need for interhemispheric interactions, leading to decreased coordination between the hemispheres. These results imply that the number of meanings in words shapes interhemispheric dynamics during visual word recognition. The implications of these findings are discussed in relation to theoretical models of visual word recognition and hemispheric differences in language processing.

## Introduction

Our brain is divided into two hemispheres: the left hemisphere and the right hemisphere. These two halves consistently interact and play distinct yet complementary roles in processing visual words, particularly at higher levels of cognitive function, such as semantic analysis. Research has demonstrated that both hemispheres contribute to the semantic processing of words (Abernethy and Coney, 1990; Perrone-Bertolotti et al., 2013; Stephan et al., 2007; Vigneau et al., 2011). For instance, when interpreting sentence sequences, it is essential to grasp the meaning of each word in relation to the context both within and between sentences. Achieving this understanding necessitates considering the various meanings of each word in the given context, which facilitates a clear and accurate interpretation of the sentence. This intricate process underscores the importance of both hemispheres collaborating to navigate the complexities of language comprehension.

However, existing models of semantic processing—such as the Multiple Access Model and the Interactive Activation Model—primarily center on lexical-semantic activation within a unified cognitive architecture, without explicitly delineating the distinct contributions of the left and right hemispheres in visual word recognition. These frameworks tend to emphasize the processes by which meaning is accessed from orthographic input, largely treating the brain as a functionally homogeneous system. Yet, a growing body of evidence suggests that the two cerebral hemispheres exhibit differential specialization in semantic processing: the left hemisphere is often associated with rapid, focused activation of dominant meanings, while the right hemisphere is implicated in broader, more diffuse semantic activation and the processing of less frequent or contextually derived meanings (Chiarello, 1985; Jung-Beeman, 2005). Despite these empirical findings, current theories have yet to fully integrate hemispheric asymmetries into formalized accounts of semantic access and representation. Thus, it is critical to characterize not only the independent roles of each hemisphere in semantic processing but also the nature of their interaction—whether cooperative, competitive, or compensatory—during visual word recognition.

For these reasons, understanding how the hemispheres process visual words with varying meanings is crucial for gaining insights into semantic processing during language comprehension. The current study aimed to explore the interactions between the two hemispheres when dealing with words that have multiple meanings in isolated word recognition. To investigate this, the study employed a visual half-field presentation paradigm (Kim and Nam, 2024), a well-established method in cognitive neuroscience for examining hemispheric processing (Bourne, 2006). In this paradigm, the target stimulus is briefly presented (for less than 200 ms) either in one of the unilateral visual fields (left or right parafoveal presentation) or in both visual fields simultaneously (bilateral parafoveal presentation). The short presentation time ensures that the stimulus is initially processed by the contralateral hemisphere due to the anatomical crossing of visual pathways (e.g., Bonandrini et al., 2023). For example, when a target is presented in the left parafoveal field, it initially activates the right hemisphere for processing. Conversely, a target in the right parafoveal field activates the left hemisphere. In the case of simultaneous bilateral parafoveal presentation, both hemispheres are activated concurrently, facilitating interhemispheric communication.

In the current study, we utilized the visual half-field presentation paradigm to examine two primary behavioral indicators. The first indicator is the right visual field advantage, which reflects superior recognition—both faster and more accurate—for words presented in the right parafoveal field compared to those in the left parafoveal field. Previous research has consistently demonstrated this right visual field advantage in the general population, which predominantly exhibits left-hemispheric language lateralization (Barca et al., 2011; Brysbaert et al., 1996; Mohr et al., 2007). This phenomenon can be attributed to the left hemisphere’s role in supporting the right visual field advantage, as suggested by the neuronal architecture of the human visual pathway (Bourne, 2006). The right visual field advantage served as a validation measure to ensure the reliability of the parafoveal projection in the current experiment. The second indicator is the bilateral redundancy gain, which indicates superior recognition (faster and more accurate) for words presented bilaterally in both left and right parafoveal fields, compared to unilateral presentation in either the left or right field (Kim et al., 2022a, 2022b; Mohr et al., 2007). According to Hebb’s cell assembly theory, the simultaneous activation of neurons across both hemispheres during word production, comprehension, or the perception of faces and objects leads to the formation of strongly connected neuronal circuits (Hebb, 1949). When familiar stimuli are presented bilaterally, these neuronal assemblies activate rapidly, leading to a fast summation of neuronal activity and the ignition of the entire assembly. Behaviorally, this results in faster and more accurate stimulus perception. The current study highlighted the significance of bilateral redundancy gain in understanding hemispheric cooperation during visual word processing and aimed to examine the mechanisms behind semantic processing and the role of interhemispheric communication.

To examine the relationship between semantic processing and interhemispheric interactions, several models have been proposed regarding the semantic processing of words. According to the Multiple Access Model (Onifer and Swinney, 1981; Swinney, 1979) and the Interactive Activation Model (Rayner and Duffy, 1986), when a word with multiple meanings is encountered, potential meanings are initially activated in parallel. This simultaneous activation occurs independently of contextual cues, facilitating the early stages of word recognition by enabling rapid access to the word’s possible interpretations. This broad activation increases the likelihood of quickly identifying the appropriate meaning, particularly in ambiguous or multi-interpretational contexts. As the recognition process unfolds, contextual information begins to exert its influence, guiding the selection of the most contextually relevant meaning. At this stage, the process may shift from facilitation to inhibition, with irrelevant meanings being suppressed to refine the word’s interpretation. This transition from facilitative to inhibitory processing is critical for the precise resolution of meaning within a given context. In scenarios of isolated word recognition, where contextual information is absent, the activation of multiple meanings can be more facilitative than inhibitory, potentially leading to faster recognition of words with multiple meanings compared to those with a single meaning. The degree of facilitation observed in reaction times (RTs) reflects the extent to which multiple meanings contribute to recognition.

Regarding the hemispheric asymmetry in differential processing based on the number of word meanings, Kim and Nam (2023a) suggest that the hemispheres use different strategies for activating multiple meanings, leading to different facilitation effects depending on how the hemispheres recognize words. Kim and Nam (2023a) investigated this phenomenon using a lateralized lexical decision task with balanced (low-frequency difference) and unbalanced (high-frequency difference) homonyms, each having only two meanings. For instance, the ambiguous word “bank” can refer either to a financial institution or the edge of a river. When these alternative meanings occur with comparable frequency, the “bank” is classified as a balanced homonym; however, if one meaning predominates significantly over the other, it is considered an unbalanced homonym. The findings revealed that responses were more accurate for unbalanced homonyms, particularly in the LVF/right hemisphere. This suggests that the right hemisphere primarily activates the dominant meaning, driven by frequency, whereas the left hemisphere simultaneously activates all candidate meanings of homonyms with comparable intensity.

This hemispheric difference aligns with previous findings on the semantic processing of ambiguous words in sentence contexts. Research has demonstrated that the left hemisphere consistently selects the contextually appropriate meaning of words with multiple meanings, regardless of their frequency (Faust and Gernsbacher, 1996; Faust and Chiarello, 1998). In contrast, the right hemisphere is more influenced by frequency of each meaning (Coney and Evans, 2000). These differences are further supported by Kim and Nam’s (2023a) study of the isolated recognition of homonyms. In their study, the left hemisphere exhibited no significant differences in responses between balanced and unbalanced homonyms, suggesting that it concurrently activates all candidate meanings of homonyms, irrespective of their frequency. This co-activation facilitates rapid selection of the contextually appropriate meaning in sentence contexts. Conversely, the right hemisphere showed differences in accuracy (ACC) between balanced and unbalanced homonyms, indicating that its processing is influenced by the frequency information associated with the meanings of homonyms.

Given these findings, the left hemisphere is expected to demonstrate stronger facilitation in the visual recognition of words with multiple meanings compared to the right hemisphere, due to its strategy of activating potential meanings concurrently. The right hemisphere’s frequency-dependent activation strategy, on the other hand, results in less robust facilitation. Furthermore, these different interhemispheric strategies may lead to varying patterns of interhemispheric interaction during visual word recognition, depending on the number of meanings a word has (Chiarello et al., 1990; Burgess and Simpson, 1988a, 1988b; Faust and Lavidor, 2003). For instance, Faust and Lavidor (2003) conducted two experiments to explore the differential hemispheric processing of words with multiple meanings. In the first experiment, they employed a primed lexical decision task where two priming words were presented that were either both related to the dominant meaning of the ambiguous word, both related to the subordinate meaning, or one related to the dominant and the other to the subordinate meaning. The results revealed that the left hemisphere demonstrated the greatest benefit from semantically convergent primes that aligned with the dominant meaning of the ambiguous word. In contrast, the right hemisphere showed the greatest benefit from semantically mixed (divergent) primes that activated alternative meanings of the ambiguous word. In the second experiment, they used the same materials but employed a semantic relatedness judgment task. This experiment demonstrated that facilitation in the right hemisphere was significantly greater when the primes were semantically mixed compared to when both primes converged on a single meaning (either dominant or subordinate) of the ambiguous word. Conversely, in the left hemisphere, facilitation occurred only when the two primes were associated with a single meaning of the ambiguous word. No facilitation was observed in the left hemisphere when the primes were semantically mixed. These findings support prior research suggesting that during word recognition, the right hemisphere activates a broader and more diverse range of related meanings, including alternative meanings of ambiguous words, compared to the left hemisphere.

In addition, Chiarello et al. (1990) examined hemispheric differences in semantic processing using an automatic semantic priming paradigm. In their study, three types of semantic relations were studies: (1) similarity-only (e.g., Deer–Pony), (2) association-only (e.g., Bee–Honey), and (3) similarity*association (e.g., Doctor–Nurse). When prime words were presented centrally, priming effects on lexical decision performance were symmetrical across visual fields for all three types of semantic relations. However, when both primes and targets were presented to the same visual field at parafoveal field, distinct patterns emerged. Specifically, similarity-only priming was greater in the left visual field (LVF) than in the right visual field (RVF), no priming effect was observed for association-only pairs, and priming for similarity*association pairs was equivalent across visual fields. These findings suggest that the right hemisphere plays a distinct role in the automatic access of semantic category relationships, particularly in processing similarity-based relations. The results also support the idea of functional asymmetries in semantic processing, with the left hemisphere rapidly selecting a dominant meaning while suppressing alternative candidates, and the right hemisphere facilitating broader, more diffuse activation of related meanings.

Based on the previous findings, words with more meanings engage more facilitative processing, particularly in the left hemisphere, because the isolated word presentation provides no context to guide the selection of the most appropriate meaning through inhibition. In lexical decision tasks, where context is absent, the inhibition mechanism is minimized, and facilitation continues throughout the early stages of visual recognition. Therefore, words with multiple meanings produce greater facilitative processing, especially in the left hemisphere, as the left hemisphere concurrently activates all candidate meanings. This facilitation is particularly strong in the left hemisphere, resulting in a greater right visual field advantage. As a result, this increased lateralization within the left hemisphere may reduce reliance on interhemispheric interactions, leading to less effective coordination between the two hemispheres and diminishing the advantage in bilateral processing.

Considering the three primary stages of visual word recognition (Dehaene et al., 2015), hemispheric processing begins with early visual processing in the visual cortex (Szwed et al., 2014). This involves the activation of V1 and V2 areas to process the physical attributes of stimuli, followed by V3 and V4 areas, which handle shape processing. Subsequently, the processing transitions to regions along the ventral visual pathway, including the visual word form area (VWFA), which processes external properties such as brightness, size, and orientation. The VWFA, located in the fusiform gyrus, is crucial for the perceptual representation of visual word letters (Cohen et al., 2003; Molko et al., 2002). After initial processing in the VWFA, phonological and lexico-semantic processing occurs in reading specific brain regions, such as the perisylvian area, utilizing the information from word letters. The differential roles of the left hemisphere and right hemisphere in processing word meaning and frequency emerge primarily during this final stage, as the earlier stages are more focused on visual-perceptual processing rather than semantic content and frequency. This suggests that the number of meanings associated with words may influence hemispheric asymmetry and interhemispheric interaction, particularly during the later stages of visual word recognition.

In the current study, we employed the ‘subjective’ meanings of words to investigate hemispheric asymmetry and interhemispheric interaction in visual word recognition, rather than relying on ‘objective’ definitions from the dictionary. This approach is based on two main considerations. First, dictionary-defined meanings do not always align with the number of meanings recognized by individual participants. For example, if a participant associates the word ‘bat’ solely with the animal and not with sports equipment, their hemispheric processing of the word will differ from that of a participant who recognizes both meanings. Second, prior research by Kim et al. (2020), which presents the data of current study, found a correlation coefficient of only 0.49 between subjective and objective word meanings, suggesting that these two measures capture distinct aspects of lexical meaning. Subjective meanings reflect individual knowledge and experiences, providing a more accurate representation of participants’ cognitive responses. While the ambiguous word “bank” can mean, for example, a financial institution or the side of a river, an individual may associate it only with the former based on personal experience. Thus, by employing ‘subjective’ meanings, we aimed to capture the individualized cognitive representations of words, allowing for a more precise examination of their influence on hemispheric processing. This approach avoids the limitations of generalized dictionary definitions, which may fail to account for individual variability.

### Two hypotheses

We proposed two hypotheses in the current study. Firstly, we hypothesized that words with multiple meanings lead to greater facilitative processing in the left hemisphere due to the co-activation of these meanings. In the context of isolated word presentation during a lexical decision task, where no contextual information is available, the inhibition of activated meanings in words with multiple meanings may be delayed or may not occur, allowing facilitation to dominate the recognition process. Furthermore, because the left hemisphere concurrently activates all candidate meanings, irrespective of their frequency (Kim and Nam, 2023a), words with more meanings are expected to generate stronger facilitation during recognition. The increased lateralization of processing within the left hemisphere likely reduces the reliance on interhemispheric interactions between the left hemisphere and the right hemisphere, potentially leading to diminished coordination between the two hemispheres. This is in contrast to the right hemisphere, which processes word meanings primarily based on their frequency (Kim and Nam, 2023a). Since the bilateral redundancy gain represents the cognitive advantage facilitated by the co-activation of both hemispheres, the increased facilitative processes in the left hemisphere caused by a higher number of meanings may inhibit the cooperative processing between the two hemispheres, leading to a decrease in bilateral redundancy gain. Thus, we expected that words with a high number of meanings would exhibit lower bilateral redundancy gain compared to words with a low number of meanings.

Secondly, given the well-established the right visual field advantage—characterized by faster and more accurate responses to stimuli presented in the RVF relative to the LVF—we hypothesized that this advantage reflects the dominant role of the left hemisphere in language processing, as RVF stimuli are directly projected to the left hemisphere (Knecht et al., 2000). We predicted that words with a greater number of meanings would elicit enhanced facilitative processing within the left hemisphere due to the concurrent activation of multiple semantic representations. This semantic convergence was expected to enhance processing efficiency—manifested as faster and/or more accurate responses—in the left hemisphere relative to the right, thereby amplifying the right visual field advantage. Consequently, we predicted that high-meaning words would yield a more pronounced RVF advantage compared to low-meaning words.

## Methods

### Participants

We reexamined the parafoveal lexical decision data from Kim et al. (2022a), which involved stimuli presented in both parafoveal and foveal vision, to explore the influence of the number of meanings of words on uni- and bi-hemispheric processing. In order to address these research questions, we performed further analyses on the data from two prior studies. For the investigation of the meanings, we utilized the scores assessed in the study by Kim et al. (2020), which involved 15 participants (7 males, 8 females) with a mean age of 26.67 ± 2.02 years. In the parafoveal lexical decision study of Kim et al. (2022a), 37 participants were initially recruited; however, one participant was excluded from the final analysis due to non-compliance with experimental protocols, resulting in a final sample of 36 participants (17 males, 19 females; mean age 23.84 ± 2.39 years). Accordingly, the total sample size across the two prior studies amounted to 53 participants. All the participants were right-handed (mean score 8.08 ± 1.82) as assessed by the Edinburgh Handedness Inventory (Oldfield, 1971). None had a medical history of neurological impairment, and all possessed normal or corrected-to-normal vision in both eyes. Kim et al. (2022a) and Kim et al. (2020) received approval from the Institutional Review Board of Korea University, South Korea, and informed consent was obtained from all participants following a thorough explanation of the ethical guidelines (KUIRB-2018-0086-01).

### Lateralized lexical decision task

**Figure 1:**
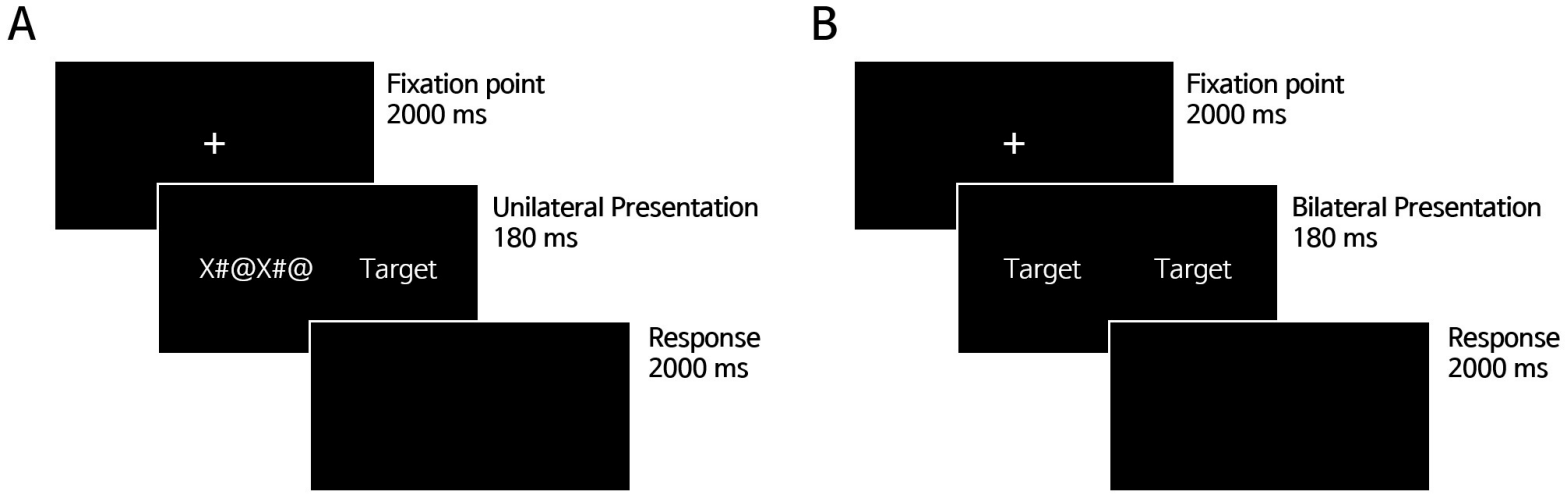
A schematic representation of the experimental paradigm (Kim et al., 2022). **(A)** Unilateral presentation of stimuli at the left or right parafoveal vision, with a string of symbols (‘X#@X#@’) at the opposite visual field used to control for visual attention in the target parafoveal vision. **(B)** Bilateral presentation of identical stimuli at both left and right parafoveal vision.

To investigate uni- and bi-hemispheric processing for Korean words, Kim et al. (2022a) employed a lateralized lexical decision task. This task involved presenting stimuli to the left and/or right parafoveal vision, leading to initial activation in the contralateral hemisphere corresponding to the unilateral visual field, thereby allowing for the assessment of lateralized and integrative hemispheric responses (Kim et al., 2020; Kim et al., 2022a, 2022b; Kim and Nam, 2023a; Kim et al., 2023a; Kim et al., 2023b; Kim and Nam, 2023b; Kim et al., 2024a; Kim et al., 2024b). The task began with a fixation point (“+”) displayed centrally on the screen for 2000 ms, followed by the presentation of a stimulus in the left, right, or both visual fields for 180 ms. Participants were instructed to maintain fixation on the central point while determining whether the presented parafoveal letter strings constituted a word or pseudoword. In the unilateral condition (left or right parafoveal presentation), a string of symbols (‘X#@X#@’) was simultaneously displayed in the visual field opposite to the target (e.g., target at LVF, symbols at RVF). A string of symbols was presented to mitigate the influence of visual attention toward the target parafoveal field. For bilateral (left and right) parafoveal presentation, identical stimuli were presented simultaneously in both visual fields without any accompanying symbol strings. Participants were given 2000 ms to respond on a blank screen after the stimulus was removed (Figure 1). They indicated their decision by pressing the slash button (“/”) for words and the “z” button for pseudowords, using the index fingers of both hands. The response key assignments were counterbalanced across participants. Specifically, half of the participants used the slash key (“/”) to indicate words and the “z” key to indicate pseudowords, while the other half did the reverse. The task included 12 practice trials before the main session of 600 trials (300 words and 300 pseudowords) started. All stimuli in the main trials were presented in a pseudo-randomized order and shown only once.

### Apparatus

Participants’ heads were stabilized with their chins on a chinrest and their foreheads against a stationary bar to ensure a fixed gaze on the screen. The stimuli were presented on an RGB-colored LG monitor.^1^ The distance between the participant’s nasion and the fixation point on the screen was maintained at 65 cm. Stimuli, consisting of white letters on a black background, were displayed within horizontal visual angles of 2° to 5° and a vertical visual angle of 1.5°. E-prime 2.0 professional software (Psychology Software Tools, Inc., Pittsburgh, PA, USA) managed the presentation and duration of the stimuli. Responses were recorded using a keyboard positioned in front of the participants.

### Materials

To construct the lateralized lexical decision task in Kim et al. (2022a), a total of 300 morphologically complex Korean words were randomly selected from diverse sources to ensure ecological validity and minimize experimental bias: internet blogs/posts (40%), books (30%), newspapers (20%), and movies (10%). The information of lexical variables for these 300 words were obtained from Kim et al. (2020), which takes a survey on semantic variables (e.g., number of objective meanings, imageability, concreteness), frequency variables (e.g., subjective familiarity, subjective frequency, stem frequency, word frequency, first syllable frequency), and length variables (e.g., number of strokes, number of letters, number of morphemes, number of phonemes). Additionally, 300 pseudowords were created by randomly combining syllables from the selected words, ensuring that they were not defined in the Korean Sejong Corpus (Kang and Kim, 2009). These pseudowords were orthographically legal and but lacked semantic meaning (Kim et al., 2025).

Kim et al. (2022a, 2022b) employed a Latin-square design to present identical stimuli across three visual field conditions: RVF, LVF, and BVF. To achieve this, they constructed three stimuli lists (List 1, List 2, and List 3), each containing 300 words and 300 pseudowords. Within each list, stimuli were equally distributed across the visual fields, with 100 words and 100 pseudowords assigned to each field. Participants were randomly assigned to one of the three lists, ensuring counterbalancing across conditions.

### Lexical variables

The present study utilized previously collected survey data on the meanings of 300 words (Kim et al., 2020). Participants were asked to subjectively rate each word based on the number of meanings, considering both homonymy and polysemy. Since we focused on examining how the subjective number of meanings influences hemispheric specialization and interhemispheric interactions, we calculated the average number of meanings for a pool of words and categorized them into two groups—150 high-meaning and 150 low-meaning words—matched for other semantic, frequency, and length variables. Consequently, the experimental condition of meanings comprised two levels: high and low. A significant difference was observed between these two levels in terms of the number of meanings [*F*(1, 298) = 7.548, *p* = 0.006, $\eta_{p}^{2}\;$ = 0.025]. Additionally, statistical analyses confirmed that other lexical variables were matched across the high- and low-SM conditions, as indicated by non-significant differences in semantic variables (number of objective meanings [*F*(1, 298) = 1.737, *p* = 0.189, $\eta_{p}^{2}\;$ = 0.006], imageability [*F*(1, 298) = 0.435, *p* = 0.510, $\eta_{p}^{2}\;$ = 0.001], concreteness [*F*(1, 298) = 0.179, *p* = 0.673, $\eta_{p}^{2}\;$ = 0.001]), frequency variables (subjective familiarity [*F*(1, 298) = 0.372, *p* = 0.542, $\eta_{p}^{2}\;$ = 0.001], subjective frequency [*F*(1, 298) = 0.015, *p* = 0.903, $\eta_{p}^{2}\;$ = 0.001], stem frequency [*F*(1, 298) = 0.087, *p* = 0.768, $\eta_{p}^{2}\;$ = 0.001], word frequency [*F*(1, 298) = 0.001, *p* = 0.994, $\eta_{p}^{2}\;$ = 0.001], first syllable frequency [*F*(1, 298) = 1.253, *p* = 0.264, $\eta_{p}^{2}\;$ = 0.004]), and length variables (number of strokes [*F*(1, 298) = 0.259, *p* = 0.611, $\eta_{p}^{2}\;$ = 0.001], number of syllables [*F*(1, 298) = 1.998, *p* = 0.159, $\eta_{p}^{2}\;$ = 0.007], number of morphemes [*F*(1, 298) = 0.509, *p* = 0.476, $\eta_{p}^{2}\;$ = 0.002], number of phonemes [*F*(1, 298) = 1.506, *p* = 0.221, $\eta_{p}^{2}\;$ = 0.005]), ensuring no confounding effects on the SM impact in the right visual field advantage and bilateral redundancy gain analyses.^2^ Table 1 shows the properties of variables that were evaluated in Kim et al. (2020). Table 2 describes the controlled lexical variables across high- and low- SM words.

**Table 1:** Description of lexical variables.

Lexical variable	Content	Value
# of strokes	Number of strokes in ‘현상을’	20
# of letters	ㅎ + ㅕ + ㄴ + ㅅ + ㅏ + ㅇ + ㅇ + ㅡ + ㄹ	9
# of syllables	현 + 상 + 을	3
# of morphemes	현상(stem) + 을(affix)	2
Stem frequency	Frequency of ‘현상(stem)’	5524
Word frequency	Frequency of ‘현상을’	814
1st syllable frequency	Number of words sharing the same first syllable (‘현’)	4942
Subjective familiarity	Degree of subjective familiarity with ‘현상을’	5.2
Subjective frequency	Frequency of exposure to ‘현상을’	4
Imageability	Degree of associating a mental image with ‘현상을’	3.2
Concreteness	Degree of sensory experience associated with ‘현상을’	2.93
# of objective meaning	Number of dictionary definitions of ‘현상을’	4
# of subjective meaning	Number of subjective meanings of ‘현상을’	1.41

The example word ‘현상을’ as a representation of lexical variable properties.

**Table 2:** Controlled lexical variables across high and low SM levels.

		Semantic variables				Frequency variables					Length variables			
		SM	Objective meanings	Imageability	Concreteness/Abstractness	Subjective familiarity	Subjective frequency	Stem frequency	Word frequency	First syllable frequency	Number of strokes	Number of syllables	Number of morphemes	Number of phonemes
Number of SMs	High	1.139 (.018)	1.593 (0.100)	4.060 (0.088)	3.839 (.109)	4.878 (0.080)	3.925 (0.068)	4771 (757)	383 (84)	7920 (546)	18.700 (0.390)	3.160 (0.042)	2.085 (025)	7.953 (0.130)
	Low	1.078 (0.012)	1.407 (0.101)	3.980 (0.083)	3.776 (0.102)	4.813 (0.072)	3.937 (0.066)	4427 (886)	382 (73)	8778 (539)	18.967 (0.350)	3.247 (0.044)	2.111 (0.026)	8.173 (0.124)

Bracketed values represent standard errors. SM, subjective meaning.

### Statistical analyses

To assess the right visual field advantage in RTs and accuracy (ACC) between words and pseudowords, we applied a linear mixed-effects regression model for RTs and a generalized linear mixed-effects regression model for ACC. The models incorporated fixed effects for lexicality (word, pseudoword) and visual half-field (VHF: RVF, LVF), while random effects accounted for variability across participants and items. Similarly, to evaluate the bilateral redundancy gain between words and pseudowords, a linear mixed-effects model for RTs and a generalized linear mixed-effects model for ACC were used, incorporating fixed effects of lexicality and bilateral redundancy gain (BVF, RVF), alongside random effects for participants and items.

In addition, we examined right visual field advantage for high versus low SM words using linear mixed-effects models for RTs and generalized linear mixed-effects models for ACC. Fixed effects included SM (high, low) and VHF, with random effects for participant and item variability. Bilateral redundancy gain was also compared between high and low SM words using analogous models for both RTs and ACC. These models included fixed effects for SM and bilateral redundancy gain, with random effects accounting for variability across participants and items. All analyses were conducted in R using lme4 (Version 1.1–30; Bates et al., 2015) and lmerTest (Version 3.1.3; Kuznetsova et al., 2017). All the models described above included a random effects structure restricted to random intercepts, excluding random slopes, due to convergence issues encountered during the analysis.

## Results

RTs and ACC data were collected from the lateralized lexical decision task, with a focus on item-based analysis for statistical evaluation. No outliers were identified, as none of the participants’ RT or ACC scores deviated by more than three standard deviations across any experimental conditions. Behavioral responses (RTs and ACC) are presented in Table 3. Table 4 also provides behavioral responses in relation to the high and low meanings of words.

1. *The right visual field advantages and bilateral redundancy gains in RTs and ACC for words and pseudowords*

**Table 3:** RTs and ACC for words and pseudowords across visual fields (RVF, LVF, BVF).

			Visual fields		
			RVF	LVF	BVF
Lexicality	Words	RTs (ms)	632 (74)	697 (92)	626 (71)
		ACC (%)	85.1 (13.0)	67.7 (17.0)	93.4 (10.0)
	Pseudowords	RTs (ms)	696 (81)	694 (72)	693 (80)
		ACC (%)	82.0 (15.8)	87.0 (13.5)	85.0 (15.2)

Bracketed values represent standard deviations. RVF, right visual field; LVF, left visual field; BVF, bilateral visual field.

**Table 4:** RTs and ACC for words with high and low SM levels across visual fields (RVF, LVF, BVF).

			Visual Fields		
			RVF	LVF	BVF
Number of SMs	High	RTs (ms)	619 (69)	697 (83)	629 (76)
		ACC (%)	85.2 (12.5)	68.2 (17.2)	93.1 (10.0)
	Low	RTs (ms)	644 (78)	698 (99)	624 (66)
		ACC (%)	84.9 (13.5)	67.3 (16.9)	93.6 (10.0)

Bracketed values represent standard deviations. RVF, right visual field; LVF, left visual field; BVF, bilateral visual field.

Table 3 presents behavioral responses for words and pseudowords across visual fields (RVF, LVF, BVF), measured using RTs and ACC. To compare right visual field advantage between words and pseudowords, the analysis revealed a significant two-way interaction between lexicality and VHF [*β* = −13.907, *SE* = 1.713, *t* = −8.119, *p* < 0.001], alongside significant main effects for both lexicality and VHF [*β* = −22.755, *SE* = 2.138, *t* = −10.645, *p* < 0.001 for lexicality; *β* = −14.302, *SE* = 1.713, *t* = −8.351, *p* < 0.001 for VHF]. Simple main effects analysis indicated a significant simple main effect of VHF for words [*β* = −29.124, *SE* = 2.434, *t* = −11.960, *p* < 0.001], showing faster RTs for words presented in the RVF compared to the LVF. However, no significant simple main effect of VHF was found for pseudowords [*β* = −0.204, *SE* = 2.366, *t* = −0.086, *p* = 0.931], indicating no RT differences between RVF and LVF pseudowords. The significant main effect of lexicality reflects faster responses for words compared to pseudowords, while the VHF effect indicates overall faster responses for RVF compared to LVF.

For ACC, a generalized linear mixed-effects model with the same fixed and random effects showed a significant two-way interaction between lexicality and VHF [*β* = 0.438, *SE* = 0.024, *z* = 18.269, *p* < 0.001], as well as significant main effects for both lexicality and VHF [*β* = −0.093, *SE* = 0.036, *z* = −2.578, *p* = 0.010 for lexicality; *β* = 0.227, *SE* = 0.024, *z* = 9.493, *p* < 0.001 for VHF]. Simple main effects analysis revealed significant VHF effects for both words and pseudowords [*β* = 0.678, *SE* = 0.034, *z* = 19.800, *p* < 0.001 for words; *β* = −0.224, *SE* = 0.035, *z* = −6.413, *p* < 0.001 for pseudowords], indicating more accurate responses for words in the RVF, but less accurate responses for pseudowords in the RVF. The main effect of lexicality suggests higher ACC for pseudowords than words, and the VHF effect reflects higher ACC for RVF stimuli compared to LVF stimuli.

To further explore the bilateral redundancy gain, the analysis of RTs revealed a non-significant two-way interaction between lexicality and bilateral redundancy gain [*β* = −1.047, *SE* = 1.651, *t* = −0.634, *p* = 0.526], but showed significant main effects for both lexicality and bilateral redundancy gain [*β* = −23.748, *SE* = 2.133, *t* = −11.132, *p* < 0.001 for lexicality; *β* = −3.717, *SE* = 1.651, *t* = −2.251, *p* = 0.024 for bilateral redundancy gain]. These results indicate faster responses for words compared to pseudowords and faster responses for BVF stimuli than for RVF stimuli.

For ACC, the analysis revealed a significant two-way interaction between lexicality and bilateral redundancy gain [*β* = 0.088, *SE* = 0.023, *z* = 3.796, *p* < 0.001], along with significant main effects for lexicality and bilateral redundancy gain [*β* = −0.123, *SE* = 0.035, *z* = −3.490, *p* < 0.001 for lexicality; *β* = 0.218, SE = 0.023, *z* = 9.372, *p* < 0.001 for bilateral redundancy gain]. Simple main effects analysis demonstrated significant bilateral redundancy gain effects for both words and pseudowords [*β* = 0.311, *SE* = 0.032, *z* = 9.652, *p* < 0.001 for words; *β* = 0.139, *SE* = 0.035, *z* = 3.999, *p* < 0.001 for pseudowords]. This suggests that while both words and pseudowords yielded more accurate responses for BVF compared to RVF, words exhibited a stronger bilateral redundancy gain than pseudowords, as evidenced by the significant two-way interaction between lexicality and bilateral redundancy gain. The main effect of lexicality indicates more accurate responses for words than pseudowords, and the main effect of bilateral redundancy gain indicates more accurate responses for BVF than RVF stimuli.

2. *The right visual field advantages and bilateral redundancy gains in RTs and ACC for words with high and low SM levels*

To compare the right visual field advantage between high and low SM words, the analysis of RTs revealed a significant interaction between SM and VHF [*β* = −7.179, *SE* = 2.491, *t* = −2.882, *p* = 0.004], alongside a significant main effect of VHF [*β* = −14.070, *SE* = 1.712, *t* = −8.219, *p* < 0.001]. However, the main effect of SM was non-significant [*β* = −2.237, *SE* = 3.318, *t* = −0.674, *p* = 0.501]. Simple effects analysis of the interaction indicated significant VHF effects for both high and low SM words [*β* = −36.580, *SE* = 3.289, *t* = −11.120, *p* < 0.001 for high SM; *β* = −21.694, *SE* = 3.594, *t* = −6.036, *p* < 0.001 for low SM]. This suggests that although both high and low SM words exhibited faster responses in the RVF compared to the LVF, the effect was more pronounced for high SM words due to the significant interaction.

For ACC, a generalized linear mixed-effects model showed no significant interaction between SM and VHF [*β* = −0.011, *SE* = 0.032, *z* = −0.337, *p* = 0.736], and no main effect of SM [*β* = 0.004, *SE* = 0.050, *z* = 0.083, *p* = 0.934]. However, there was a significant main effect of VHF [*β* = 0.246, *SE* = 0.023, *z* = 10.577, *p* < 0.001], indicating greater ACC for responses in the RVF compared to the LVF.

To compare bilateral redundancy gain between high and low SM words in terms of RTs, the analysis revealed a significant two-way interaction between SM and bilateral redundancy gain [*β* = 6.913, *SE* = 2.296, *t* = 3.011, *p* = 0.003], alongside a significant main effect of bilateral redundancy gain [*β* = −3.905, *SE* = 1.650, *t* = −2.367, *p* = 0.018]. However, the main effect of SM was not significant [*β* = −2.968, *SE* = 3.332, *t* = −0.891, *p* = 0.373]. Simple effects analysis of the interaction showed a significant bilateral redundancy gain effect for low SM words [*β* = −11.951, *SE* = 3.286, *t* = −3.637, *p* < 0.001], but no significant bilateral redundancy gain effect for high SM words [*β* = 2.094, *SE* = 3.072, *t* = 0.682, *p* = 0.495]. This indicates that low SM words exhibited faster responses in the BVF compared to the RVF, while high SM words showed no significant difference in response times between BVF and RVF. The significant main effect of bilateral redundancy gain suggests overall faster responses for BVF compared to RVF.

**Figure 2:**
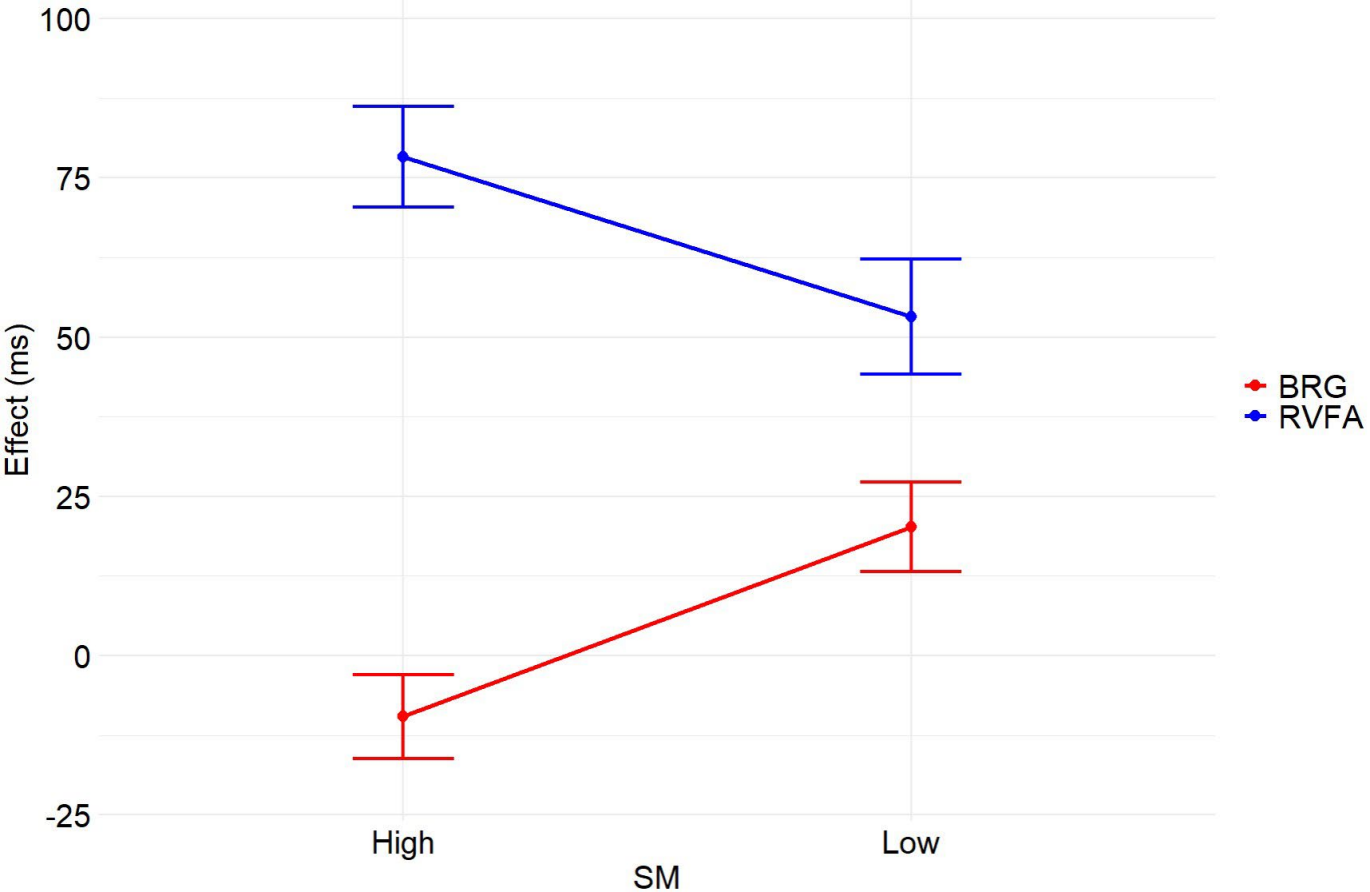
The RVFAs and BRGs in RTs for words with high and low SM levels. Error bars represent standard error.

**Figure 3:**
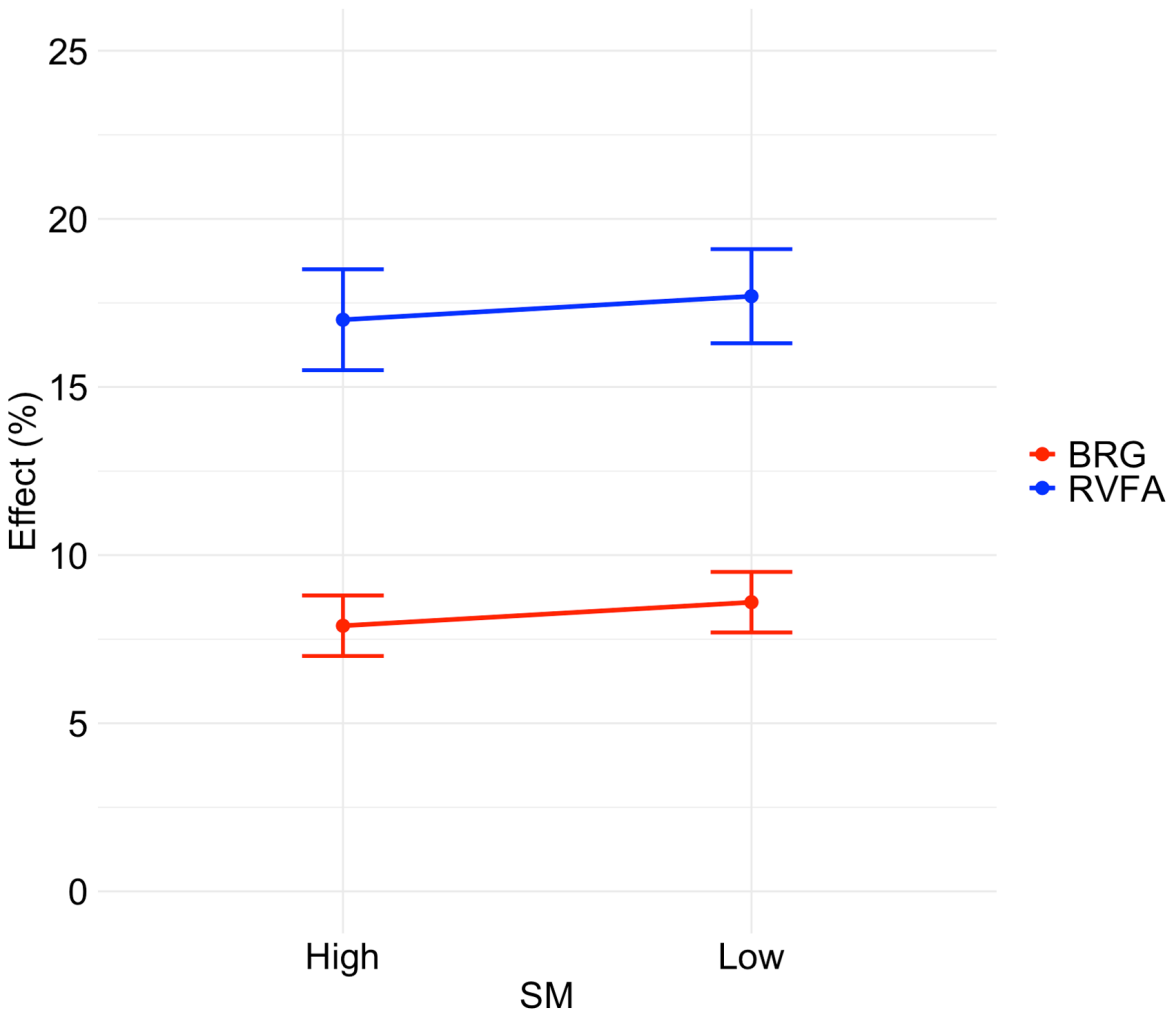
The RVFAs and BRGs in ACC for words with high and low SM levels. Error bars represent standard error.

Additionally, for ACC, the analysis showed no significant interaction between SM and bilateral redundancy gain [*β* = −0.012, *SE* = 0.032, *z* = −0.363, *p* = 0.717], and no significant main effect of SM [*β* = 0.004, *SE* = 0.050, *z* = 0.078, *p* = 0.938]. However, there was a significant main effect of bilateral redundancy gain [*β* = 0.224, *SE* = 0.023, *z* = 9.652, *p* < 0.001], indicating more accurate responses for BVF compared to RVF. Figures 2, 3 illustrate the right visual field advantages and bilateral redundancy gains in RTs and ACC for words with high and low SM levels.

## Discussion

On the parafoveal lexical decision task, for words there was a significant positive right visual field advantage in RTs and ACC, and a significant positive bilateral redundancy gain in ACC. Conversely, for pseudowords, there was a significant negative right visual field advantage in ACC and a significant positive bilateral redundancy gain in ACC. These result agree with previous evidence (e.g., Mohr et al., 2007) that performance for words is generally better in the RVF than in the LVF and better in the BVF than in the RVF. On the other hand, pseudowords tend to perform worse in the RVF relative to the LVF and exhibit minimal improvement in the BVF compared to the RVF. The non-significant right visual field advantage in RTs for pseudowords suggest that pseudoword processing, unlike word-processing is not associated with lateralized hemispheric processing in the left hemisphere, in contrast to word processing. This alignment with prior studies suggests that the data analysis design in the current study was effectively implemented, thereby ensuring reliable interpretation of the findings.

Moreover, the study revealed diminished bilateral redundancy gain and greater right visual field advantage in RTs for words with a high number of meanings compared to words with a low number of meanings. This effect was not observed in ACC, indicating that the influence of the number of meanings is specific to processing speed (RTs) rather than ACC in visual word recognition. The reduced bilateral redundancy gain observed for words with high meanings can be attributed to facilitated responses for stimuli presented in the RVF, rather than in the BVF, suggesting that the reduced interhemispheric interaction is primarily driven by the left hemisphere. In the left hemisphere, the multiple meanings of words tend to facilitate each other, leading to faster responses for RVF stimuli. Additionally, the weaker right visual field advantage in RTs for words with a low number of meanings, compared to those with a high number of meanings, was due to slower responses in the RVF rather than enhanced responses in the LVF. These results imply that the left hemisphere’s management of multiple meanings significantly impacts processing efficiency, as reflected in the observed patterns of bilateral redundancy gain and right visual field advantage.

The new findings of this study indicate that the number of meanings associated with words significantly influences interhemispheric interactions during visual word recognition. Building on the previous research of Kim and Nam (2023a), it is evident that the left hemisphere is actively involved in activating potential meanings during visual word processing. This concurrent activation of multiple meanings is expected to facilitate the recognition process, as each meaning contributes to the efficient selection and recognition of the word. In contrast, the right hemisphere adopts a different approach, activating meanings with varying intensities based on their frequency—with more frequent meanings receiving stronger activation. This frequency-driven activation in the right hemisphere results in minimal facilitation among the meanings, as the dominant meaning is naturally prioritized according to its frequency. Consequently, the left hemisphere, unlike the right hemisphere, is more prone to facilitative processes arising from the simultaneous activation of multiple meanings, enhancing its efficiency in processing words with a high number of meanings. This finding from the current study aligns with and the work of Chiarello et al. (1990), who demonstrated that the right hemisphere contributes uniquely to the automatic access of semantic category relationships, particularly in processing similarity-based associations. Their findings suggest functional asymmetries in semantic processing: the left hemisphere appears to rapidly select the dominant meaning of a word while actively suppressing alternative interpretations, whereas the right hemisphere supports a broader, more diffuse activation of semantically related meanings. As observed in the current study, there is a lower bilateral redundancy gain when recognizing words with a high number of meanings compared to those with fewer meanings. The diminished bilateral redundancy gain for high-subjective-meaning words highlights the left hemisphere’s challenge in managing multiple competing meanings, which in turn affects the overall efficiency of interhemispheric cooperation during visual word recognition tasks.

Previous research has proposed the coarse coding hypothesis, which posits that the left hemisphere selectively activates the contextually appropriate meaning of a word, while the right hemisphere engages a broader spectrum of meanings based on semantic relatedness (Beeman, 1998; Jung-Beeman, 2005). According to this hypothesis, during the recognition of polysemous words, the left hemisphere is involved in a targeted process of activating potential meanings to identify the one that aligns best with the context. This mechanism is crucial for pinpointing the most contextually relevant meaning within a given sentence or discourse. Conversely, the right hemisphere does not focus on selecting contextually pertinent meanings but rather generates a wide array of meanings based on their semantic proximity and frequency of use. The right hemisphere’s expansive activation approach implies that meanings are activated according to their general prevalence; thus, more frequently encountered meanings receive stronger activation, while less frequent ones are less emphasized. This strategy enables the right hemisphere to maintain a diverse range of potential meanings without prioritizing contextually appropriate selections. Consequently, the different left and right hemisphere activation strategies may result in enhanced facilitative processes within the left hemisphere when processing polysemous words. Given that the left hemisphere activates possible meanings simultaneously to determine the most appropriate one, this concurrent activation increases the likelihood of facilitation among meanings, thereby improving the efficiency of meaning selection. In contrast, the right hemisphere’s reliance on frequency-based activation yields reduced facilitation and fewer facilitative processes, as meaning selection is more heavily influenced by frequency. This distinction between the left hemisphere’s selective activation and the right hemisphere’s broad activation elucidates why the left hemisphere might exhibit stronger facilitative processes during the recognition of multiple-meaning words.

### Theoretical models

Several theoretical models support the findings of the current study. The Dual Route Cascaded model (Coltheart et al., 2001) posits two distinct pathways: a lexical route that processes familiar words through stored lexical knowledge and a non-lexical route that decodes unfamiliar words or pseudowords by converting graphemes to phonemes. This model aligns with the observed hemispheric differences, suggesting that the lexical route corresponds with the left hemisphere’s processing of familiar words and meanings, while the non-lexical route parallels the right hemisphere’s processing based on frequency and general patterns. The Dual Route Cascaded model supports the notion that hemispheric interactions vary with word familiarity, reinforcing the study’s findings that interhemispheric differences are influenced by the number of meanings a word possesses, underscoring the role of “subjectiveness” in word processing. Additionally, the Interactive Activation Model (McClelland and Rumelhart, 1981) proposes that word recognition involves parallel processing and interactive feedback across multiple levels—features, letters, and words. According to Interactive Activation Model, activation propagates from visual features to letters to words, with feedback mechanisms aiding in the resolution of ambiguities associated with polysemous words. This model suggests why the left hemisphere exhibits enhanced facilitation for high SM words, as interactive feedback helps reconcile competing meanings, thereby promoting more efficient processing and resulting in reduced bilateral redundancy gain. Connectionist models, particularly the Parallel Distributed Processing approach (Plaut et al., 1996), further examine these findings by proposing that word recognition involves distributed networks of interconnected units representing orthographic, phonological, and semantic information. The facilitative effects observed in the left hemisphere can be attributed to competitive interactions among these units, with facilitation emerging from the activation of multiple meanings. Conversely, the right hemisphere’s frequency-dependent activation aligns with how connectionist models weight connections based on experience, leading to more efficient processing but less facilitation for polysemous words. The current study’s results concerning the right visual field advantage and bilateral redundancy gain underscore the significance of hemispheric strategy in visual word recognition. The reduced bilateral redundancy gain observed for words with a high number of meanings suggests that the left hemisphere may benefit from preparing each potential meaning for selection. This heightened lateralization of the processing in the left hemisphere likely reduces the need for interhemispheric interactions between the left hemisphere and right hemisphere, leading to diminished coordination across hemispheres. In contrast, the right hemisphere’s frequency-dependent activation minimizes such facilitation, resulting in less efficient recognition.

### Strengths of the current study

The current study has several strengths. Firstly, it utilized subjective measures rather than objective ones to determine the number of word meanings. Objective measures, such as those derived from corpora (Kang and Kim, 2009), could present potential problems for this investigation since we aimed to explore variations in participants’ hemispheric interactions based on the number of word meanings. Therefore, using subjective measures to evaluate the number of meanings of words provides a more reliable understanding of the changes in interhemispheric interactions in visual word recognition. Secondly, the current study identified significant changes in interhemispheric interaction at the behavioral level in visual word recognition. By employing the visual half-field presentation paradigm, we investigated interhemispheric interactions and evaluated the bilateral redundancy gain as a behavioral indicator of the advantage of interhemispheric interactions. Our findings revealed a significant decrease in bilateral redundancy gain for words with a high number of meanings compared to words with a low number of meanings. This significant behavioral finding strongly suggests that there are notable variations at the neurological level in interhemispheric interactions in visual word recognition depending on the number of meanings of words. These results suggest the need for future research on the neural mechanisms underlying the behavioral patterns we observed. Thirdly, we controlled for various length variables, frequency variables, and semantic variables between words with low and high numbers of meanings. By controlling these sublexical and lexical variables, the current study provides more reliable results, offering a clearer understanding of the changes in interhemispheric interactions in visual word recognition based on the number of meanings of words. Lastly, the present findings are novel, distinguishing them from those of Kim et al. (2022a), from which the data were derived. While Kim et al. (2022a) focused on the alteration of interhemispheric interactions in relation to word familiarity, the current study makes a significant contribution by demonstrating how interhemispheric interactions are modulated by the number of meanings associated with words. These findings are expected to advance our understanding of the hemispheric mechanisms involved in visual word processing, particularly with regard to interhemispheric communication.

Although it suggests significant implications, one potential concern is that lexical decisions in the current task may be driven predominantly by orthographic familiarity, with participants relying on the visual familiarity of words relative to pseudowords rather than engaging in deeper lexical or semantic analysis. According to previous studies, the right hemisphere has been shown to exhibit heightened sensitivity to visual familiarity due to its reliance on perceptual learning mechanisms and holistic pattern recognition processes (Cohen and Dehaene, 2004; Dehaene et al., 2010). This familiarity-based processing enables the right hemisphere to activate multiple lexical or semantic representations when encountering visually familiar inputs, consistent with the findings suggesting that familiarity operates as a frequency-related variable in modulating activation breadth (Kim et al., 2020). In contrast, the left hemisphere, which is more specialized for sublexical/lexical processing for visual words (Joubert et al., 2004), potentially exhibits relatively reduced sensitivity to visual familiarity. As a result, the left hemisphere is hypothesized to activate lexical representations in a more categorical and selective manner, leading to a more uniform activation across meanings regardless of their familiarity. Another potential confound in the interpretation of our results stems from the morphological complexity of the visual word stimuli. However, the experimental paradigm employed in this study specifically probed semantic processing rather than relying on morphological processing. Given that morphological decomposition typically occurs at the sublexical or early lexical stages of word recognition, its influence on higher-level processes like semantic analysis is presumed to be diminished. Furthermore, to mitigate any residual impact of morphological decomposition on our findings, we controlled for a comprehensive suite of linguistic variables. These included various length variables (number of strokes, syllables, morphemes, and phonemes), frequency variables (subjective familiarity, subjective frequency, stem frequency, and word frequency, alongside first syllable frequency), and semantic variables (number of objective meanings, imageability, and concreteness). By matching of these variables across words with both low and high numbers of meanings, we substantially minimized the potential for morphological decomposition effects to confound our results.

### Limitations of the current study

There are limitations to this study despite its significant implications. The first pertains to the ‘subjective’ data regarding the number of meanings assigned to each word in the materials. In this study, ratings of the subjective number of meanings were obtained from a different sample than the participants who performed the lateralized lexical decision task. As a result, the subjective nature of the data is not perfectly aligned with the participants involved in the primary task. Addressing this issue would require the same participants to perform both the lexical decision task and provide subjective meaning ratings. However, this presents a practical challenge, as the variables across experimental conditions cannot be manipulated prior to the experiment without prior knowledge of how participants rate the meanings of each word. Consequently, this study partially acknowledges the limitation of subjective measures. Nonetheless, the subjective ratings used here offer a more accurate reflection of individual cognitive responses compared to dictionary-derived objective meanings, which fail to generalize to individual experiences effectively. A second limitation of our study is that the averaging of subjective ratings risks obscuring individual-level variations. This averaging may mask actual participant-specific subjective interpretations. For instance, if a participant predominantly associates the word ‘bat’ with the animal rather than sports equipment, their hemispheric processing of that word could differ significantly from an individual who subjectively recognizes both meanings. Therefore, future research should integrate trial-level or participant-specific meaning ratings, potentially employing a mixed-effects modeling, to directly investigate how subjective semantic representations influence hemispheric processing. The third limitation concerns the nature of interhemispheric interactions revealed in this study. While our investigation revealed the advantages of bilateral interactions for words with a low number of meanings compared to those with a high number of meanings, it did not examine the exact nature of the interhemispheric interaction. Previous research has indicated that interhemispheric interaction could occur in specific directions, such as from the left hemisphere to the right hemisphere, from the right hemisphere to the left hemisphere, or bidirectionally. For instance, Kim et al. (2023a) utilized Granger causality analyses on EEG data to examine interhemispheric interactions during visual word recognition in relation to word familiarity. Their analyses showed a stronger transfer of information from the right hemisphere to the left hemisphere during the N100 processing stage and a weaker transfer from the left hemisphere to the right hemisphere during the N400 processing stage for highly familiar word recognition. This suggests that the directionality of interhemispheric interaction might vary depending on the specific characteristics of the task or stimuli. Therefore, it is plausible that there is a specific pattern of interhemispheric interaction in visual word recognition that varies based on the number of meanings a word has. To study these interactions more precisely in order to reveal how interhemispheric dynamics change with the number of meanings in words, future studies should employ neuroscientific methods such as Granger causality or other advanced EEG/fMRI techniques. A final limitation of the present study concerns the potential influence of orthographic familiarity on participants’ lexical decisions. Given that the task involved both words and pseudowords, there remains the possibility that participants relied primarily on the visual familiarity of the stimuli—rather than engaging in deeper lexical analysis—when making their judgments. Visually familiar stimuli may have elicited a stronger subjective sense of word-likeness, leading participants to respond “word” to pseudowords that conformed to familiar orthographic patterns. To mitigate this potential confound, we employed pseudowords that adhered to the orthographic rules of the language, rather than nonwords, and carefully matched subjective familiarity across experimental conditions for the word stimuli. The design was intended to reduce the likelihood that orthographic familiarity alone could account for the observed effects. Nonetheless, the possibility remains that familiarity-based processing contributed to task performance, and thus, the interpretation of the results should be made with caution, taking into account the potential contribution of orthographic familiarity to the observed patterns.

Additionally, although the primary aim of the study was not to investigate sex differences in interhemispheric interactions or hemispheric lateralization, we conducted supplementary analyses incorporating sex as a fixed factor within the established models for bilateral redundancy gain and right visual field advantage, applied to both RTs and ACC. These analyses revealed no significant interactions involving sex for either RTs or ACC,^3^ suggesting that sex did not modulate interhemispheric dynamics and lateralized processing of visual words with multiple meanings in the current dataset. Nonetheless, given prior evidence linking sex differences to cerebral specialization (Boles, 2005; Gur and Gur, 2017), it remains possible that sex may influence language-related hemispheric asymmetries under different task conditions or paradigms, requiring constant consideration of sex variable in hemispheric studies.

## Data Availability

The datasets presented in this study can be found in online repositories. The names of the repository/repositories and accession number(s) can be found in the article/supplementary material.

## Footnotes

^1^The clarity of the presented stimuli was augmented by the use of an LG monitor, a product of the South Korean electronics firm recognized for its high-quality displays. Further contributing to visual precision, the stimuli were presented against a Red, Green, Blue (RGB) color background, directly correlating with the primary colors utilized in back-lighting technology. This combination of advanced display hardware and optimized chromatic presentation was critical in maximizing stimulus visibility and distinctness.

^2^The semantic, frequency, and length variables were already evaluated in Kim et al. (2020). For an in-depth examination of each variable, please refer to the appendix of Kim et al. (2020).

^3^Linear mixed-effects regression analyses of RTs revealed no significant three-way interaction between bilateral redundancy gain, subjective meanings (SMs), and sex (*p* = 0.144), nor between right visual field advantage, SMs, and sex (*p* = 0.675). Similarly, generalized linear mixed-effects regression analyses of ACC yielded non-significant interactions for both bilateral redundancy gain × SMs × sex (*p* = 0.144) and right visual field advantage × SMs × sex (*p* = 0.144).
